# Impact of Coronavirus Disease 2019 Outbreak on Children and Adolescents with Type 1 Diabetes Mellitus

**DOI:** 10.34172/jrhs.2021.67

**Published:** 2021-11-14

**Authors:** Tayebeh Hasan Tehrani, Zahra Razavi, Samin Salimi, Hamidreza Farahi, Hasan Bazmamoun, Ali Reza Soltanian

**Affiliations:** ^1^Mother and Child Care Research Center, Hamadan University of Medical Sciences, Hamadan, Iran; ^2^Department of Pediatrics, School of Medicine, Hamadan University of Medical Sciences, Hamadan, Iran; ^3^Islamic Azad University, Tabriz Branch, Tabriz, Iran; ^4^Modeling of Noncommunicable Diseases Research Center, Hamadan University of Medical Sciences, Hamadan, Iran; ^5^Department of Biostatistics, School of Public Health, Hamadan University of Medical Sciences, Hamadan, Iran

**Keywords:** COVID-19, Diabetic Ketoacidosis, Glycemic Control, Type 1 Diabetes Mellitus

## Abstract

**Background:** This study aimed to investigate the impact of the Coronavirus Disease 2019 (COVID-19) pandemic on daily routines and health of patients with type 1 diabetes mellitus (T1DM).

**Study design:** A cross-sectional study.

**Methods:** This study included 98 children and adolescents with type 1 diabetes who were regularly followed up in the Endocrinology outpatient clinic of Besat Hospital, Hamadan, Iran, in 2020. The primary measurements included body mass index, glycemic control, number of hypoglycemic events, number of hospitalizations, as well as bedtime and availability of insulin six months pre and post COVID-19 outbreak. The obtained data were analyzed in SPSS software (version 16). A p-value less than 0.05 was considered statistically significant.

**Results:** Out of 98 participants (mean ±SD age: 13.5±49), 51% of the cases were male, and %81.6 of the patients were urban residents. Furthermore, most participants (43.9%) were in the age group of 11-15 years, and the mean ±SD duration of diabetes was 4.64±3.31 years. In addition, 2.04% of the participants developed COVID-19. There was a statistically significant difference among average duration of night sleep (*P*<0.001), bedtime (*P*<0.001), time of waking up (*P*<0.001), amount of insulin intake (*P*=0.003), daily exercise (*P*<0.001), and availability of the insulin (*P*<0.001) before and during COVID-19 crisis. The frequencies of hospitalizations and hypoglycemic events were lower after the COVID-19 outbreak (*P*=0.005 and *P*=0.034, respectively). Morning insulin dose was omitted in 22.2% of participants. No differences were found between hemoglobin A1c and daytime sleep pre and post COVID-19 outbreak.

**Conclusions:** The key challenges due to COVID-19 pandemic restrictions for Iranian T1DM patients were the need to take more insulin, lower physical activity, non-availability of insulin, and missed morning insulin dose. However, it is worth mentioning that the patients' blood glucose control did not worsen in this period.

## Introduction

In early December 2019, an outbreak of severe acute respiratory syndrome caused by a novel severe acute respiratory syndrome coronavirus 2 (SARS-CoV-2), which was later determined to be coronavirus disease 2019 (COVID-19) emerged in Wuhan (Hubei, China) and rapidly increased globally ^1-3^. It was declared a pandemic by the World Health Organization on March 11, 2020^4^. The emergence of the outbreak of COVID-19 has caused a global public health emergency. On February 19, 2020, the first case of the disease was announced in Iran. To prevent the spread of COVID-19, the restrictions included home confinement, and nose/mouth coverings in confined public areas, as well as the closure of most places, such as gyms, swimming pools, primary and secondary schools, and higher education institutions. These restrictions continued throughout the survey period.

The global pandemic of COVID-19 disease led to unprecedented economic, psychological, and health challenges in many countries and regions of the world ^5^. The restrictions that were imposed to prevent the transmission of the disease and the behavior of COVID-19 may have a negative impact on patients with chronic diseases^6^, including those with diabetes mellitus. There is no conclusive evidence that individuals with well-controlled type 1 diabetes are at risk for COVID19 disease^7^. However, the International Association of Children and Adolescents Diabetes has commented on continuing optimal diabetes care during the COVID-19 crisis in order to prevent referral to emergency care or hospitalization^8^. On the other hand, lockdown, fear of developing the COVID-19, and provision of health and medical services to patients with COVID-19 may affect the access of diabetic patients to medications, medical services, or health facilities. Therefore, the routine and necessary care of these patients may be neglected during this period.

 The burden of this pandemic is less addressed on vulnerable populations with type 1 diabetes in Iran with a high incidence of the COVID-19. This study aimed to investigate the impact of COVID-19 pandemic on metabolic control, routine life, and health of children and adolescents with type 1 diabetes mellitus (DMI). Moreover, it attempted to evaluate the challenges this population faced in Hamadan, Iran. The identification of the patients' challenges leads to the development of strategies to maintain the health of diabetic patients during these greatest challenges faced by humankind in the recent century.

## Methods

 The study protocol was approved by the Ethics Committee of Hamadan University of Medical Sciences, Hamadan, Iran (IR.UMSHA.REC.1399.442.). Informed written consent was obtained from parents and patients before participation in the study. This descriptive cross-sectional study was conducted in the first half of 2020 on 98 patients with type 1 diabetes diagnosed before the age of 19 years who received different insulin regimens for ≥12 months. Patients were recruited from the outpatient diabetes clinic at Besat Hospital, Hamadan, Iran, in 2020. This center is a tertiary care hospital that provides diabetes care to approximately 600 children with DM1.

The primary measurements included glycemic control through measuring hemoglobin A1c (HbA1c), mean of fasting and postprandial blood glucose (mg/dl), weight (kg), total daily dose insulin (unit/kg/day), number of insulin injections per day, number of hypoglycemic events, number of episodes of diabetic ketoacidosis, insulin dose missed, availability of insulin, access to medical services or health facility, sleep routine (bedtime, average duration of night sleep, duration of daily sleep), number of blood glucose measurements per week, daily physical activity, number and cause of hospitalizations, and insulin availability six months before and during the COVID-19 outbreak. Required information regarding age, gender, level of parent's education, place of residence, parents' occupational status, age at diagnosis, duration of diabetes, insulin regimen (twice daily, multiple doses of insulin injection [MDI], continuous subcutaneous insulin injection system [CSII]), development of COVID-19 disease, as well as concerns about developing COVID-19 disease in patients and their families were also collected. The data were obtained from questionnaires through face-to-face interviews with parents and patients. Details of other required variables, including weight, BMI, mean blood glucose, and HbA1c were extracted from patients' medical records. The samples were divided into four age groups of 0-4, 5-10, 11-15, and 16-20 years. A "Depression, Anxiety and Stress Scale-21 Items" was used to assess participants' stress levels ^9,10^. Average blood glucose and HbA1C in the lockdown were compared with the readings before the COVID-19 crisis.

 Inclusion and exclusion criteria: ‏This study included all patients‏ diagnosed with type 1 diabetes for at least 12 months and received at least two daily insulin‏ injections and were followed up at the Endocrine outpatient clinic in Besat Hospital, Hamadan, Iran. On the other hand, the patients with concomitant use of medications other than insulin or agents that interfere with glucose metabolism, and those who showed the emergence of any new chronic diseases six months before and during the COVID-19 outbreak were excluded from the study.

 Descriptive results were expressed as mean ±standard deviation (SD), and categorical variables were presented as percentages. Differences between pre and post COVID-19 lockdown were compared through paired t-test, Kappa coefficient, Chi-square test, and McNamara test. The obtained data were entered and analyzed in SPSS software (version 16). A p-value less than 0.05 was considered statistically significant.

## Results

A total of 98 cases were included in this study. The mean ±SD age of the participants was 13.5 ±4 years, and the majority (51%) of the cases were male. It is worth mentioning that 81.6% of the patients were urban residents, and most participants (43.9%) were in the age group of 11-15 years. The mean ±SD duration of diabetes was 4.64 ±3.31 years, and 95% of participants were on MDI insulin therapy consisting of four injections including one injection of long-acting insulin in the evening and an injection of rapid-acting insulin before each meal per day. Moreover, one patient was on CSII. A total of 2.04% of the participants developed COVID-19 disease. The encounter characteristics of the included cases are summarized in Table 1. There was a statistically significant difference among the average duration of night sleep (*P*<0.001), bedtime (*P*<0.001), time of waking up (*P*<0.001), amount of insulin intake (*P*=0.003), daily exercise (*P*<0.001), and presence at the gym (*P*<0.001) before and during the COVID-19 outbreaks.

**Table 1:** Characteristics of the entire study population (n=98)

**Continuous variables**	**Mean**	**SD**
Disease duration (yr)	4.64	3.31
Age (yr)	13.56	4.94
**Categorical variables**	**Number**	**Percent**
Age (yr)		
<5	2	2.1
6-10	25	25.5
11-15	43	43.8
>15	28	28.6
Gender		
Male	48	49.0
Female	50	51.0
Residential region		
Urban	80	81.6
Rural	18	18.4
Father's education level		
Illiterate	7	7.1
Elementary	35	35.7
Diploma	27	27.6
Academic	29	29.6
Mother's education level		
Illiterate	6	6.1
Elementary	49	50.0
Diploma	28	28.6
Academic	15	15.3
Father's occupational status		
Worker	16	16.3
Employee	26	26.5
Self-employed	50	51.0
Retired	6	6.1
Mother's occupational status		
Housewife	7	7.1
Employed	91	92.9

Furthermore, a statistically significant difference was observed in the availability of insulin before and during COVID-19 (P<0.001). The frequencies of hospitalizations and hypoglycemic events were significantly lower after the COVID-19 outbreak (P=0.005 and P=0.034), respectively. In addition, no differences were found in the mean fasting blood glucose, the mean two hours postprandial blood glucose, HbA1c, daily sleep time, frequency of blood glucose measurements per week, as well as access to physicians and health services before and during COVID-19 outbreaks. Although average blood glucose during restrictions was higher than the time before COVID-19, the difference was not statistically significant (P=0.150). Morning insulin omission was reported by nearly a quarter of respondents (22.2%) due to a delay in waking up. About half of patients reported some degree of concern about developing COVID-19 disease in themselves and their families. In this regard, 44.9% of the cases had severe stress. Moreover, 70% of the participants were upset about the closure of schools and staying home, and the key source of their dissatisfaction was that they required taking classes and exams online. Table 2 shows a comparison of data measured before and during the COVID-19 crisis.

**Table 2:** Descriptive data of the analyzed samples

**Continuous variables**	**Before the COVID-19 epidemic**			**During the COVID-19 epidemic**			***P*** **-value**
	**Mean**	**SD**	**95% CI**	**Mean**	**SD**	**95% CI**	
HbA1c	9.12	2.34	8.65, 9.58	9.40	2.31	8.94, 9.86	0.149
Fasting blood glucose (mg/dl)	187.67	89.05	169.82, 205.52	186.69	81.51	170.34, 203.03	0.900
2 hours post prandial blood glucose (mg/dl)	181.17	101.53	160.81, 201,52	198.45	108.63	176.67, 220.23	0.180
Body mass index (kg/m^2^)	19.76	4.62	18.83, 20.69	20.19	4.73	19.24, 21,14	0.002
Daily insulin doses (unit/kg/day)	0.83	0.32	0.76, 9.89	0.88	0.30	0.82, 0.94	0.003
Hypoglycemic events (time/week)	1.27	1.72	0.92, 1.68	0.97	1.62	0.64, 1.29	0.034
Duration of night sleep (hour/night)	8.09	1.31	7.83, 8.35	9.10	1.65	8.76, 9.43	0.001
Duration of daily sleep (hour/day)	0.72	1.32	0.44, 0.98	0.72	1.37	0.44, 0.99	0.527
Blood sugar measurements (number/ week)	14.25	10.41	12.16, 16.33	13.08	10.41	10.99, 15.16	0.177
Hospital admission (time/6months)	0.38	0.60	0.26, 0.50	0.13	0.44	0.04, 0.21	0.005
Daily activity (hour/day)	2.32	0.74	2.17, 2.47	1.91	0.88	1.73, 2.09	0.001
**Categorical variables**	**Number**	**Percent**	**95% CI**	**Number**	**Percent**	**95% CI**	***P*** **-value**
Easy access to insulin	98	100	82.93, 94,27	13	13.2	5.73, 17.69	0.001
Bedtime							
10-12 pm	51	52.0	48.19, 68.67	47	47.9	42.46, 63.18	0.001
After 12 pm	31	31.6	24.93, 44.73	67	68.7	66.32, 84.24	0.001
Wake up time							
Before 8 am	77	77.8	79.42, 93.63	32	32.3	25.99, 45.92	0.001
8-11 am	22	22.2	15.76, 33.67	42	42.8	36.82, 57.56	0.001
After 11 am	0	0.0	0.00, 0.00	22	22.2	15.76, 33,68	0.001
Insulin omission	0	0.0	0.00, 0.00	22	22.2	15.76, 33,63	0.001

## Discussion

 The current COVID-19 pandemic has posed an unprecedented burden on global public health. This study focused on evaluating the impact of this overburdened crisis on lifestyle, health, and blood glucose control in subjects with T1DM after the closure of all educational institutions and suspension of sports activities in Iran. The results of this study demonstrated that the closure of schools and colleges along with the restrictions in outdoor activities to limit the spread of COVID-19have changed the patients' routines. Our data showed a significant increment in daily insulin dose, BMI, duration of night sleep, interrupted insulin supply, and bedtime followed by a decrease in daily activities. However, glycemic control has not been worsened. The data of this study revealed that the average duration of night sleep was significantly longer during the COVID-19 crisis than the time before the outbreak.

Prior studies have shown that inadequate sleep may affect self-management behaviors and glycemic control of patients with T1DM ^11,12^. According to a study conducted by Patel NJ et al, a significant association was found between sleep duration and poorer metabolic control. In this regard, they concluded that the modification of the quantity and quality of sleep might improve metabolic control status^13^. Furthermore, Ji X. et al. highlighted the importance of sleep education in the care program for children, adolescents, and young adults with T1DM^14^. Increased night sleep duration could lead to more relaxation during the day and prevent glycemic control deterioration in our patients^15^. Since insulin consumption was higher during the COVID-19 crisis than before this pandemic, the lack of deterioration in glycemic control may be attributed to increased insulin intake.

Another notable finding of this study was that the frequencies of hypoglycemia and hospitalization were lower than those before the COVID-19 outbreak. As other researchers have suggested^16,17^, fear of getting this disease can affect patients' self-awareness leading to better management in order to maintain and improve good glycemic control, particularly because diabetes has been reported in the media as a risk factor for getting COVID-19 disease that worsens the prognosis. Therefore, despite the detrimental psychological effects of the COVID-19 crisis, either of the aforementioned factors may have prevented the worsening of blood glucose control in the studied population. Consistent with the findings of our study, Capaldo et al. in a study conducted in Italy found that during COVID-19 lockdown, 35% of adult participants with T1DM had increased sleep duration without a statistically significant difference in blood glucose target range ^18^.

Recent studies from different European countries, including Italy, Greece, and the UK, show an improved glycemic control in children, adolescents, and adults with T1DM during COVID-19 crisis^18-25^. The authors suggest that the allocation of more time for self-management, a decrease in work-related distress, a delay in routine daily activities, and a more stable rhythm of life during the lockdown may have beneficial effects on glycemic control and T1DM management at least in the short term. ^12,16,20^. Our data show that BMI increased during the COVID-19 crisis. This can be attributed to decreased daily activity and increased sleep duration. Weight gain may be one of the reasons for the lack of improvement in blood glucose control in our study, compared to studies conducted in Europe^18-25^. This result was similar to that in a study conducted by Al Agha in Saudi Arabia ^26^. Similarly, in a study by Marigliano et al. in Verona (Italy), after three months of lockdown, females had a higher risk of weight gain ^21^. The results of our study are consistent with the findings of a study performed by Tornese et al. (Italy 2020) and Ludvigsson et al. (Sweden 2020) who found that glycemic control in adults with T1DM did not deteriorate during the lockdown of COVID-19 pandemics^27,28^. In contrast to our findings, Verna et al. (India 2020) has demonstrated deterioration of glycemic control in Indian patients due to a lack of access to insulin/glucostrip, decreased levels of exercise, and non-adherence to the meal plan ^7^. According to the results of a study by Ghosal et al. (India 2020), the duration of the lockdown was proportional to the deterioration of glycemic control and the onset of diabetes-related complications^29^.

Among the participants, two (2.04%) patients developed COVID-19 disease that did not lead to hospitalization, and they fully recovered. This finding confirms the results of previous studies claiming that COVID-19 infection in pediatric patients seems to be clinically less severe than that in adults^30^. To date, there is no evidence that T1DM subjects have a higher risk for COVID-19 infection^31-32^. It is hypothesized that the course of COVID-19 disease is even milder in patients with type 1 diabetes which may be partly justified by the imbalance of Th1 versus Th2 in these patients ^33^.

 In this study, 22.2% of patients reported waking up at 11 a.m. and not injecting morning insulin. However, their blood glucose control was not worse than before. None of the patients reported a lack of access to outpatient clinics, health centers, and health care providers. This finding was to be expected because all health centers continue to provide routine services and care to chronic patients during the COVID-19 crisis. One of the most ongoing problems for our patients was limited access to insulin owing to global sanctions and limits on money transfers via global banks at the same time as the pandemic. Lack of access to insulin was a major concern for patients and parents. Surveillance and planning are needed to facilitate the delivery of this medication to minimize the negative impact of diabetes and the COVID-19 crisis on patients and their families.

Our findings highlight that many of our patients were dissatisfied with closing schools and colleges, followed by staying home. They expressed their tension and anxiety as a result of having to take classes and tests online. However, in a study in India ^7^, most patients were happy to stay home with their parents. The inconsistency in this data may be mainly due to the fact that more time has passed since the schools have been closed in our study, and therefore, our patients are tired.

 Regarding the limitations in this study, the recruited participants had regular follow-up at the diabetes clinic. Therefore, our findings cannot be generalized to the entire T1DM population in Iran. Moreover, no data have been available on changes in the meal plans or the amount of food intake due to the closure of schools, colleges, restaurants, and fast food stores (unhealthy diet). Finally, the survey period may not be long enough to provide an accurate assessment of the psychological impact, lifestyle changes, and challenges that children and adolescents with T1DM are facing during the current pandemic. Further studies with a larger sample size and longer follow-up periods may reveal different results. Nevertheless, this is a preliminary study conducted early in the COVID-19 outbreak and provides information that could be useful to health care workers and contribute to the advancement of future research.

## Conclusion

 Restrictions and school closures to prevent the spread of COVID-19 changed the patients' routines. The key challenges due to COVID-19 pandemic restrictions for Iranian T1DM patients were the need to take more insulin, lower physical activity, increased BMI, non-availability of insulin, and missed morning insulin dose. Despite this, a significant deterioration of glycemic control and diabetes-related complications were not reported in this study. All outpatient clinics were available and continued to provide services to patients. The frequency of hypoglycemia and hospitalization was lower during COVID-19 pandemic restrictions.

## Acknowledgments

 The authors would like to express their gratitude to all the participants and their parents attending the Diabetes clinic and contributing to the completion of this study. They also thank the Clinical Research Center of Besat Hospital, Hamadan University of Medical Sciences, Hamadan, Iran.

## Funding

 The authors received no funding from an external source and declare no conflicts of interest.

 Highlights

- During the COVID-19 epidemic limitations, Iranian T1 diabetic patients required more insulin, and they had less physical activity, as well as access to insulin.
- Patients and their families could properly manage the disease in the critical condition of the COVID-19 pandemic.
- COVID-19 crisis did not exert a negative impact on glycemic control of patients with type 1 diabetes mellitus at least in the short term.
